# Thermoresponsive Lignin-Reinforced Poly(Ionic Liquid) Hydrogel Wireless Strain Sensor

**DOI:** 10.34133/2021/9845482

**Published:** 2021-12-07

**Authors:** Xinyu Qu, Ye Zhao, Zi'ang Chen, Siying Wang, Yanfang Ren, Qian Wang, Jinjun Shao, Wenjun Wang, Xiaochen Dong

**Affiliations:** ^1^Key Laboratory of Flexible Electronics (KLOFE) and Institute of Advanced Materials (IAM), School of Physical and Mathematical Sciences, Nanjing Tech University (NanjingTech), Nanjing 211816, China; ^2^School of Physical Science and Information Technology, Liaocheng University, Liaocheng 252059, China

## Abstract

To meet critical requirements on flexible electronic devices, multifunctionalized flexible sensors with excellent electromechanical performance and temperature perception are required. Herein, lignin-reinforced thermoresponsive poly(ionic liquid) hydrogel is prepared through an ultrasound-assisted synthesized method. Benefitting from the electrostatic interaction between lignin and ionic liquid, the hydrogel displays high stretchability (over 1425%), excellent toughness (over 132 kPa), and impressive stress loading-unloading cyclic stability. The hydrogel strain sensor presents excellent electromechanical performance with a high gauge factor (1.37) and rapid response rate (198 ms), which lays the foundation for human body movement detection and smart input. Moreover, owing to the thermal-sensitive feature of poly(ionic liquid), the as-prepared hydrogel displays remarkable thermal response sensitivity (0.217°C^−1^) in body temperature range and low limit of detection, which can be applied as a body shell temperature indicator. Particularly, the hydrogel can detect dual stimuli of strain and temperature and identify each signal individually, showing the specific application in human-machine interaction and artificial intelligence. By integrating the hydrogel strain sensor into a wireless sensation system, remote motion capture and gesture identification is realized in real-time.

## 1. Introduction

The flourishment of wearable devices has demonstrated huge applicable potential in intelligent terminals, bionic electronic skin, prosthetic replacement, and health monitoring, which motivated extensive research interests in flexible electronics [1–4]. The flexible sensors, with critical requirements to attach to the surface of various shapes and realize multiple stimuli perception in a wide range of deformations, are the most important components in flexible wearable devices [5, 6]. Therefore, compared to traditional rigid sensors, flexible sensors are under rigorous demands to perform superior flexibility, stretchability, sensitivity, and conformality [7–10]. At present, many elastic substrates, such as polyurethane, polydimethylsiloxane, and silicone rubber, have been commercialized with advantages of easy processing, facile preparation, and satisfying tensile properties [8, 11–13]. Specifically, by incorporating conductive fillers into the flexible substrates, highly accurate and sensitive electromechanical perception is achieved in the flexible strain sensors [14–17]. However, the inherent toxicity and poor stretchability in those commercialized flexible substrates limit their application to organisms and restrict their precisely tactile perception of humidity and temperature [18–21]. In general, there lays a pressing demand for a flexible electronic device with tunable mechanical properties, splendid biocompatibility, and multimodal response.

The hydrogel, with a unique three-dimensional (3D) hydrophilic polymer network, performs high stretchability, exceptional biocompatibility, and degradability, exert broad application prospects in the fields of biological wound dressings, drug delivery, sensors, actuators, and flexible energy storage devices [22–27]. Ionic liquids are a group of organic salts comprised of organic cations and organic or inorganic anions [28, 29]. They are chemical and thermally stable, biocompatible, and antibacterial and can maintain fluidity at or near room temperature [30–32]. By composing the ionic liquids into hydrogels (ionic gel), the low saturated vapor pressure and high ionic conductivity of ionic liquids would endow the hydrogel with a highly sensitive perception at a wide temperature range [30, 33, 34]. Moreover, since the numbers of the migratable ions and the ion migration rate vary with the temperature, the ionic gel displays a distinct thermal-sensitive feature, being feasible for multimodal signal sensation [29, 30, 33]. In a typical strategy, the ion gel is fabricated through a solvent exchange method by soaking the hydrogel into ionic liquids [34]. For example, Zhao et al. synthesized poly-2-acrylamido-2-methyl-1-propanesulfonic acid (PAMPS) ion gel by radical crosslinking polymerization of AMPS and alpha-ketoglutaric acid under UV initiation and following infiltration in 1-ethyl-3-methylimidazolium dicyanamide ([EMI] [DCA]) ionic liquid [35]. PAMPS ion gel was manifested with high transparency and stretchability, while its elongation at break was merely 120%. Sun et al. dissolved 3-dimethyl (methacryloyloxyethyl) ammonium propane sulfonate and acrylic acid (AA) into a mixture of [EMI] [DCA] ionic liquid and water and achieved ion gel with high ionic conductivity (1.1 mS cm^−1^) and antifatigue resistance [36]. However, the ion gel displayed unsatisfactory stretchability of 800%, originated from the poor interaction between the polymer matrix and ionic liquids. In addition, the direct introduction of ionic liquids into the hydrogel 3D frameworks through a straight dipping process or one-pot method cannot strictly prohibit the leakage of ionic liquids and inevitably brings detrimental effects on the tensile property of the ion gel [33, 36]. Poly(ionic liquid) hydrogel is a sort of ion-conductive polymer network copolymerized from ionic liquids and various organic monomers. It inherits the high ionic conductivity, chemical stability, and biocompatibility of ionic liquids and reveals high fluid retention ability to avoid ionic liquid leakage [29, 32, 35]. Moreover, incorporating specific-functionalized monomers in poly(ionic liquid) has also been a promising candidate for high-performance multimodal ion gel.

Lignin is a kind of complicated phenolic natural organic polymer that exists widely in nature. The abundant phenolic hydroxyl groups can form extensive physical and chemical interactions, such as metal coordination, hydrogen bonding, and ionic interaction, to greatly enhance the stretchability and hydrophilicity of the hydrogel [8, 37–40]. For example, Wang et al. polymerized 3,4-ethylene dioxythiophene monomers on the surface of sulfonated lignin (SL) with the addition of AA and Fe^3+^ to the suspension to form a strongly interacting Fe-SL hydrogel [40]. The chelating coordination between the phenolic hydroxyl group of SL and Fe^3+^ enabled Fe-SL hydrogel outstanding stretchability of 1680%, while the excessive physical interactions also depressed the tensile strength to 40 kPa. Inspired by the biological tissues with toughness diversity in different components, it is conducive to introduce lignin or its derivatives as the functionalized monomers into poly(ionic liquid) to construct a polymer network with both “soft” and “hard” segments, achieving promotion in both mechanical performance and perception sensitivity.

Herein, lignin-reinforced poly(ionic liquid) hydrogel was fabricated by the ultrasound-assisted synthesized method. Lignin in the branched chains of poly(ionic liquid) greatly enhanced the hydrophilic property and mechanical and electrical performance of the hydrogel. The synthesized poly(ionic liquid) hydrogel revealed excellent stretchability (up to 1425%), toughness (over 132 kPa), and cyclic stability. The strain sensor based on the poly(ionic liquid) hydrogel displayed high sensitivity (GF = 1.37) and short response time (198 ms) for human body movement detection and human-machine interaction. Furthermore, attributing to the thermal-sensitive feature of ionic liquid, the poly(ionic liquid) hydrogel can be utilized as a body shell temperature indicator, providing reliable assistance for monitoring and tracking human health status. By integrating the hydrogel sensor into a wireless sensation system, real-time human motion can be perceived, showing great potential in the fields of motion monitoring, sports posture correction, and postinjury rehabilitation.

## 2. Results and Discussion

### 2.1. Preparing and Characterization of Poly(Ionic Liquid) Hydrogels

**Figure 1:**
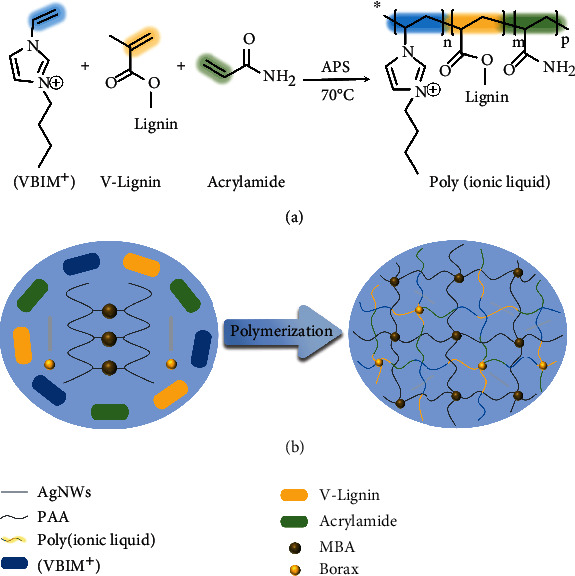
Schematic illustration for the design of poly(ionic liquid) hydrogel. (a) The copolymerization process of ionic liquid, vinyl-modified lignin, and acrylamide. (b) Schematic illustration for the preparation mechanism of poly(ionic liquid) hydrogel.

Thermoresponsive lignin-reinforced poly(ionic liquid) hydrogel with interpenetrating binary-networked nanoarchitectures was prepared by ultrasound-assisted radical polymerization followed by double-network fabrication. As shown in Figure 1, 1-vinyl-3-butylimidazolium bromide ([VBIM^+^] Br^−^) ionic liquid, vinyl-modified lignin (v-lignin), acrylamide (AM), borax, ammonium persulfate (APS), and distilled water were mixed uniformly and polymerized with the assistance of ultrasound to obtain poly(ionic liquid). Afterward, the poly(ionic liquid) was immersed in a solution of acrylic acid (AA), silver nanowires (AgNWs), *N,N*′-methylene bisacrylamide (MBA), and APS to crosslink and form a second polymer matrix. The introduction of vinyl-modified lignin with abundant catechol and pyrogallol groups in the poly(ionic liquid) chain segment considerably improves the mechanical and hydrophilic properties of the hydrogel. Tremendous phenolic hydroxyl groups in lignin form toughly covalently crosslinking with borax, and electrostatic interactions manipulated from the negatively charged lignin and the positively charged [VBIM^+^] ionic liquid further enable apparent promotion in mechanical property of the hydrogel. Moreover, the polymeric ionic liquid copolymer and the second soft and brittle polyacrylic acid (PAA) network, establish “hard” and “soft” segments entangled polymer matrix. The synergistic effect of the heterostructure facilitates rapid stress conduction and energy dissipation, which motivates crucial enhancement in the stretchability and toughness of the hydrogel [41].

**Figure 2:**
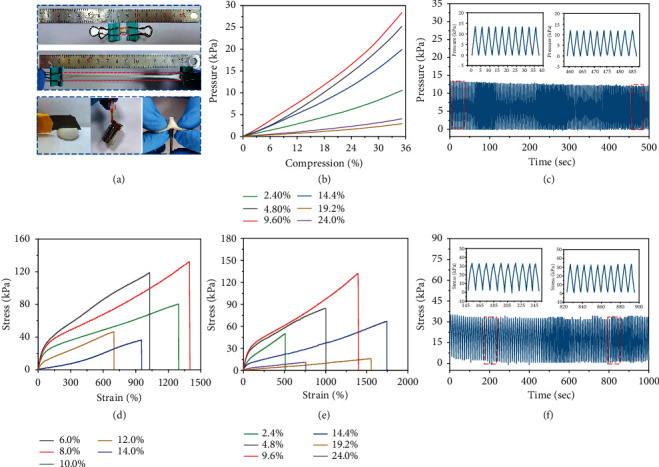
Mechanical properties of poly(ionic liquid) hydrogel. (a) Diagrams for the hydrogel at stretching, loading 200 g weight, knife pressing, and puncturing. (b) Pressure-compression curves of the hydrogel with different ionic liquid content. (c) Compressive cycle stability of the hydrogel at 20% compression strain. (d, e) Stress-strain curves of the hydrogel with different ionic liquid and lignin contents, respectively. (f) Stretching cycle stability of the hydrogel at 100% tensile strain.

Benefitting from the interwoven “soft” and “hard” segments in the double-network hydrogel, the poly(ionic liquid) hydrogel presents high stretchability and toughness. As shown in Figure 2(a), it can be stretched to more than 10 times the original length without damage and recover to its initial shape after relaxation. With exceptional resilience, the poly(ionic liquid) hydrogel can be curled, knotted, and stretched after knotting without fracture (Figure S1). Figure S2 demonstrates the ten consecutive compression loading-unloading cycles of the poly(ionic liquid) hydrogels under 14% compression. The small hysteresis loops during cycles suggest effective energy dissipation under deformation, and negligible compression residual can be distinguished at each hysteresis loop, showing a prominent resilience of the poly(ionic liquid) hydrogel. The poly(ionic liquid) hydrogel also presents excellent resistance to local stress concentration. It can easily lift a 200 g weight with a strip and resist sharp objects scraping or puncturing (such as knife, scissors, and tweezer) with negligible cracks or scratches. Moreover, due to the abundant catechol groups in lignin, the poly(ionic liquid) hydrogel characterizes a certain degree of adhesion to tightly anchor to various surfaces of stone, PTFE, and metals to afford more elaborate perception (Figure S3).

With abundant chemical and physical interaction in the polymer matrix, poly(ionic liquid) hydrogel exhibits impressive mechanical properties. Figures 2(b) and 2(e) demonstrate the influence of the contents of ionic liquid and v-lignin on the poly(ionic liquid) hydrogel mechanical properties. It appears that the compressive strength gradually increases with the increasing ionic liquid content. When the content of the ionic liquids reaches 9.6%, the compressive strength reaches a maximum of 27 kPa. However, the compressive strength decreases dramatically with further enhancement of ionic liquid content. The tensile strength experiences a similar trend in Figure 2(e), and the poly(ionic liquid) hydrogel proceeds the maximum toughness of 132 kPa at the weight ratio of 9.6% with a superior stretchability of 1425%. The nanoscaled pore size distribution, in close correlation with the crosslink density, plays a fundamentally important role in directing energy dissipation under deformations and shows a crucial effect on regulating the mechanical performance of the hydrogel. With the increase of ionic liquid content, stronger interchain and intrachain electrostatic interactions are achieved associated with higher crosslink density. And the hydrogel average pore size and the breadth of the pore distribution in hydrogel decrease dramatically [42]. Hence, the synergistic effect from the ascended crosslink density in the double network, the descending pore size, and narrower pore size distribution endow the poly(ionic liquid) hydrogel with an optimal ion liquid content. Therefore, the hydrogels with an ionic liquid content of 9.6% are chosen for follow-up experiments.

Similarly, the effect of the content of modified lignin on the mechanical properties of the poly(ionic liquid) hydrogel is evaluated in Figure 2(d). Modified lignin is incorporated into the long chain of poly(ionic liquid), and the abundant phenolic hydroxyl groups from lignin establish covalent bonds through borax and hydrogen bonds with carboxyl to crosslink the poly(ionic liquid) and PAA long chains into a highly interweaved polymer matrix. However, with a sustainable increase in v-lignin content, the excessive crosslinking will depress the hydrogel to be brittle and fragile, deteriorating the mechanical performance of the hydrogel. As shown in Figures 2(d) and S4, the stretchability and toughness of the poly(ionic liquid) hydrogel strike the optimum balance when the modified lignin content reaches 8%.

To elucidate the durability and mechanical stability of the poly(ionic liquid) hydrogel, tensile and compressive loading-unloading cycle tests are performed. As shown in Figure 2(c), the poly(ionic liquid) hydrogel can maintain stable mechanical resistance more than 100 times at a 20% compression ratio. Meanwhile, the poly(ionic liquid) hydrogel can also sustain stable mechanical reliance at a 100% stretching ratio for more than 100 stretching-releasing cycles. The poly(ionic liquid) hydrogel, with high stretchability and excellent compression properties, provides a promising prospect for wearable flexible electronic devices.

### 2.2. Electromechanical Properties of the Poly(Ionic Liquid) Hydrogels

**Figure 3:**
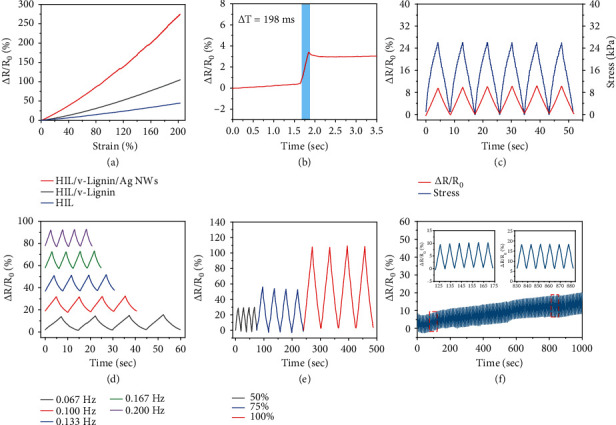
Electrical performances of the poly(ionic liquid) hydrogel strain sensor. (a–c) Sensitivity, response time, and electromechanical hysteresis of the sensor. (d) Relative resistance variations under cyclic stretching-releasing with different stretch frequencies. (e) Relative resistance variations at 50%, 75%, and 100% tensile strain. (f) Electrical cycle stability of the sensor.

Electromechanical response sensitivity is a fundamental indicator to evaluate the performance of hydrogel strain sensors. Gauge factor (GF), calculated from formula GF = (Δ*R*/*R*_0_)/*ε*, is introduced to measure the sensitivity of the sensor, where *R*_0_ is the initial resistance, Δ*R* is the relative resistance, and *ε* is the applied strain. The AgNW content was selected as 0.3%, based on the previous research [23]. Figure S5 displays the SEM images of the AgNWs which present roughly 1.5-2.5 *μ*m in length and 30-40 nm in diameter.  Figure 3(a) illustrates that AgNWs play a crucial role in enhancing the sensitivity of the poly(ionic liquid) hydrogel strain sensor. AgNWs are uniformly distributed in the poly(ionic liquid) hydrogel network and construct a highly conductive network. Moreover, the v-lignin, interacting with ionic liquid branched chains to architect a second ionic conductive network, plays a role in promoting rapid ion transfer to prompt the sensing performance. When the tensile strain is less than 200%, the relative resistance change is linear with the GF about 1.37. In Figure 3(b), it can be distinguished that poly(ionic liquid) hydrogel exerts a short response time of nearly 198 ms to tiny deformation, ensuring a rapid response rate to external stimuli.  Figure 3(c) shows the electrical signal output of the poly(ionic liquid) hydrogel strain sensor to consecutive mechanical deformations of 30% strains. The relative resistance variations are generally synchronized with the strains. The negligible electromechanical hysteresis ensures excellent replicability and reliability of the poly(ionic liquid) hydrogel strain sensor.

Figure 3(d) presents the output electrical signals of the poly(ionic liquid) hydrogel strain sensor with different frequencies under 25% tensile strain. It illustrates that the poly(ionic liquid) hydrogel strain sensor displays different time constants with diverse frequencies. And the relative resistance variation is consistent at different loading rates, exhibiting high reliability to various forms of mechanical deformations.  Figure 3(e) demonstrates the electrical response of the sensor to various tensile strains for consecutive four cycles. It appears that the relative resistance variations are 29.5%, 53.9%, and 108.3% at 50%, 75%, and 100% tensile strain, respectively. These results are consistent with the GF values in Figure 3(a) and suggest high feasibility for different ranges of human movement detection. Moreover, to sincerely evaluate the stability of the poly(ionic liquid) hydrogel strain sensor, Figure 3(f) performs the current response of the sensor under cyclic tension loading and releasing at 20% strain. The results show that the relative resistance variations exhibit a delicate response to tension loading and unloading. They remain stable during 100 consecutive stretching-releasing cycles, indicating impressive stability and durability of the poly(ionic liquid) hydrogel strain sensor. However, the hydrogel exhibited a water loss with a ratio of 23% after being exposed at 20°C and 66% RH for 24 h (Figure S6). The loss of water leads to an increase in the conductivity of the hydrogel, which may be the main reason for the drift of the electrical baseline during the cyclic stretching process.

### 2.3. Application of Flexible Touch Panel and Human Movement Detection

**Figure 4:**
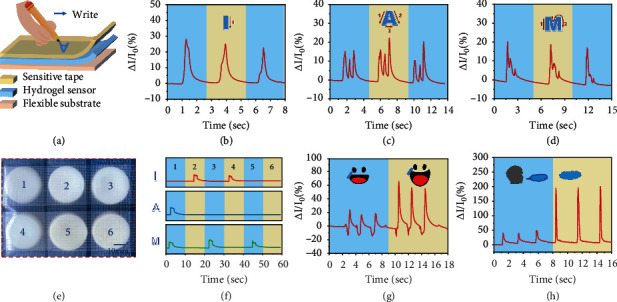
Poly(ionic liquid) hydrogel strain sensor for flexible touch panel and human movement detection. (a) Schematic illustration for the flexible touch panel. (b–d) Characteristic signal peaks for writing different letters. (e) Flexible touch panel array for braille input system. (f) Electromechanical response of typing different letters by the braille input system. (g) Relative current variations of the sensor for mouth opening. (h) Relative current variations of the sensor for blowing.

The high sensitivity and rapid response rate of poly(ionic liquid) hydrogel strain sensors have widely extended their potential in flexible touch panels and wearable flexible sensors.  Figure 4(a) schematically demonstrates the configuration of a flexible touch panel. The poly(ionic liquid) hydrogel strain sensor is sandwiched between two pieces of flexible film, where the top layer is a piece of sensitive tape to support and encapsulate the hydrogel, and the bottom layer is another piece of VHB tape serving as the flexible substrate. During the test, letters with different strokes and writing rules are selected. In Figure 4(b), the letter “I” displays only one stroke and one featured peak is revealed. In comparison, the letter “A” presents three featured peaks of different intensities for three strokes (Figure 4(c)). For the letter “M,” the response signal (Figure 4(d)) is more complicated for distinct intensity diversity in stroke writing, and continuously, featured peaks are obtained to express consecutive movements in the second stroke. It is clear that writing actions with diversity in direction, speed, and intensity present differentiable electrical signals in the flexible touch panel. Therefore, the poly(ionic liquid) hydrogel strain sensor can accurately distinguish the handwriting of different letters and even the writing habits of different people, showing huge application prospects in smart input and signature anticounterfeiting. In Figure 4(e), an array of six poly(ionic liquid) hydrogel strain sensors is assembled into a flexible touch panel to support the braille input system, where each hydrogel strain sensor corresponds to a number and different combinations of numbers refer to different letters. The input of letters and words can be sincerely realized according to the signal peaks corresponding to each key (Figure 4(f)).

The poly(ionic liquid) hydrogel strain sensor, with high stretchability and high toughness, is identified as a promising candidate for wearable flexible strain sensors to monitor various ranges of human movement. In Figure 4(g), the hydrogel is fixed on the corner of the mouth to judge the opening and closing states at speaking or making facial expressions. The relative resistance variations exhibit visible differences on the opening amplitudes of the mouth for different smiles owing to the distinct difference in the extent of muscle movement at the corners of the mouth. Meanwhile, the hydrogel fixed between the eyebrows can also accurately record the frown signal peaks (Figure S8(a)), showing great applicable potential for microexpression recognition.  Figure 4(h) demonstrates the relative current variations of the poly(ionic liquid) hydrogel strain sensor for microinterference. When blowing through the hydrogel sensor, a deep blowing is accompanied by a sharp relative current change, while a slight blowing is referred to as the gentle current signal. These phenomena demonstrate its promising prospect to monitor breath frequency and intensity before and after exercise. Apart from small-scale strain detection, the poly(ionic liquid) hydrogel strain sensors can also achieve a precise perception of large-scale body movement. As shown in Figure S8(b), the hydrogel is fixed at the finger joints to detect finger bending. When the finger is bent at different amplitudes, signal peaks of different intensities appear, providing the possibility for gesture identification.

### 2.4. Thermosensitive Performance and Application of Poly(Ionic Liquid) Hydrogels

**Figure 5:**
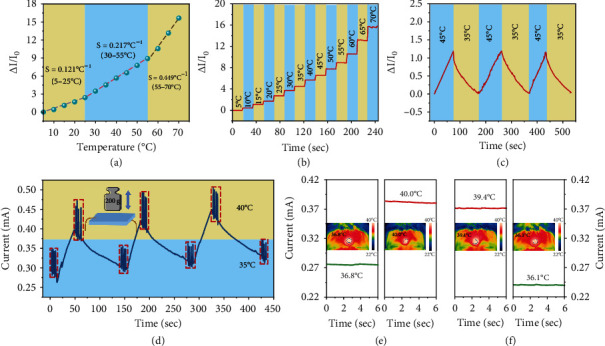
Thermosensitive performance and application of poly(ionic liquid) hydrogel. (a) Relative current changes at different temperatures. (b) The relative current changes in a temperature gradient of 5-70°C. (c) Instantaneous electrical response of the sensor to hot (45°C) and cold (35°C) source. (d) Performance of multiple stimulations of pressure and temperature. (e, f) The current changes during body fever and antipyretic process, and the inset pictures correspond to a simulated body fever.

Since the end of 2019, the new coronavirus has swept the world, causing a huge loss of life and property. As the epidemic continues to spread, new requirements have been put forward for a highly sensitive wearable temperature sensor that can attach to human skin and accurately perceive real-time temperature changes. However, most of the available hydrogel temperature sensors possess limited thermal sensitivity to distinguish the normal-fever body temperature and cannot meet the requirements of a body shell temperature indicator. The thermal-sensitive feature of ionic liquids enables the poly(ionic liquid) hydrogel sensor a prospective temperature sensor. The conductivity of the ionic liquid is in close correlation with the temperature, originating from the changes in numbers of carries and ion mobility rate at different temperatures. The temperature coefficient of current (TCC) is generally used to evaluate the thermal sensitivity of the poly(ionic liquid) hydrogel and is defined from the formula TCC = [(*I* − *I*_0_)/*I*_0_)]/Δ*T*, where *I* is the instant current of hydrogel under measured temperatures and *I*_0_ is the initial response current of the hydrogel. During the tests, the poly(ionic liquid) hydrogel was encapsulated by PU medical film tape to avoid water loss at high temperatures. The conductivity of ionic gel (*σ*) at different temperatures follows Vogel-Tamman-Fulcher (VTF) equation, *σT*^1/2^ = *A* exp (−*B*/Δ*T*), where *A* is a prereference factor and *B* is the pseudo activation energy [43].  Figure 5(a) shows the relative current variations of poly(ionic liquid) hydrogel at different temperatures. It is distinguished that with the temperature increase, the relative current variations increase sharply, showing an impressive thermal sensitivity of the poly(ionic liquid) hydrogel temperature sensor, and the curve shape is in great agreement with the VTF equation. The temperature sensor displays a wide temperature sensing range of 5-70°C. In particular, a nearly linear relationship between the relative current variations and temperature is achieved in the operating range of 25-55°C, showing an excellent thermal response sensitivity of 0.217°C^−1^. When a stepwise temperature test from 5 to 70°C is applied in Figure 5(b), distinguishable and stable relative current changes reveal at each temperature. The current response is in line with the fit curve in Figure 5(a) with a high signal-to-noise ratio, demonstrating high referability and reliability of the poly(ionic liquid) temperature sensor. Specifically, when the poly(ionic liquid) temperature sensor is exposed to repeated temperature variation at 35 and 45°C (Figure 5(c)), rapid and distinct repeated signal peaks appear, suggesting high sensitivity and excellent cycle stability to temperature deviation.

Collective detection on the poly(ionic liquid) hydrogel sensor has demonstrated that the poly(ionic liquid) hydrogel sensor can separately respond to strain, compression, or temperature; however, further studies are needed to determine its resolution to multistimuli, which to simplify its configuration in the application of human-machine interface and IntelliSense. Herein, the poly(ionic liquid) hydrogel sensor is pressed with a 200 g weight with intermittent temperature variation at 35 and 40°C. As shown in Figure 5(d), the current peak reveals intensities from 0.27 to 0.35 mA for consecutive five pressing-releasing cycles at 35°C, when the temperature rises to 40°C, the current accompanies with sharply heightened intensities from 0.37 to 0.47 mA. With further temperature divergence, the current also displays a distinct deviation in the typical sensing range. These ascents and descend cycles in current signals can be repeated to identify pressure at different temperatures, suggesting that the sensor can detect two stimuli simultaneously and identify each stimulus through different current baselines. It is noteworthy that the amplitude of the current peak shows a small deviation at each cycle, and this difference can be an indicator of the fluctuation in ambient temperature, showing high accuracy and reliability of the temperature sensor.

The excellent thermal sensitivity, immediate response rate, and high signal-noise ratio of poly(ionic liquid) hydrogel temperature sensor ensure it a precise indicator for body temperature. In Figure 5(e), the forehead was pressed by a hot towel to simulate body fever, and the hydrogel was encapsulated with PU medical film tape and fixed on the forehead, and a thermal imaging was applied to record the forehead temperature in real-time. As shown in Figures 5(e) and 5(f), within the narrow range of human body temperature, the hydrogel temperature sensor can accurately export distinct and stable current signals at different intensities, demonstrating a highly identifiable temperature resolution of 3.2°C. The accurate simulation on body fever and antipyretic process provide reliable monitoring for the early disease diagnosis and treatment.

### 2.5. Wireless Strain Sensor Based on Poly(Ionic Liquid) Hydrogels

**Figure 6:**
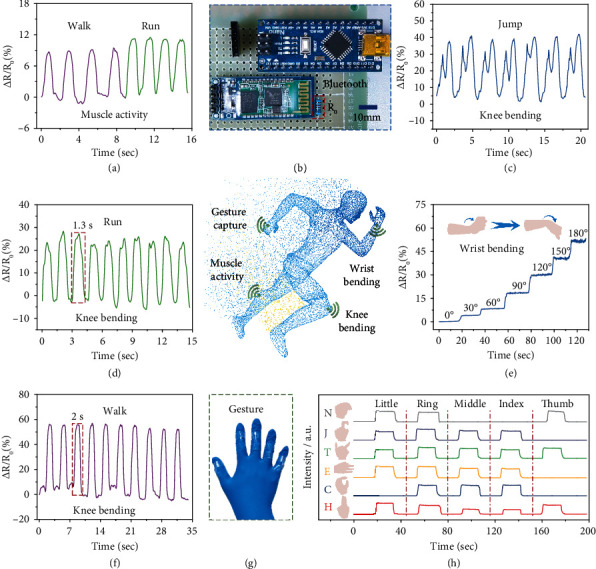
Wireless strain sensor based on poly(ionic liquid) hydrogels. (a) Response signal of calf muscle movement during running and walking. (b) Photographs of the configuration of the wireless sensation system. (c, d, f) Relative resistance changes during jumping, running, and walking under knee bending. (e) Relative resistance changes of the wrist at different bending angles. (g) Photographs of “data glove” mounted with the assembled wireless strain sensors. (h) Relative resistance changes of the data glove responding to different gestures “N, J, T, E, C, and H.”

Taken together with our aforementioned study data indicates that the poly(ionic liquid) hydrogels manifest precise perception to strain, compression, and temperature, showing prospect feasibility for the flexible wearable device. Furthermore, to demonstrate its real-time detection on human motion detection, a proof of concept prototype with a highly integrated wireless sensation system, composed of a microcontroller unit (MCU), a Bluetooth module, and a marked resistor (*R*_0_) is manufactured.  Figure 6(b) exhibits the fabricated device and its integration, which transmits signals through Bluetooth wireless communication in real time. The hydrogel strain sensor is connected in series with a 10 k*Ω* standard resistance, and the resistance value of the strain sensor (*R*_*x*_) is calculated by measuring the partial voltage (*V*_0_) on standard resistance, which calculation formula is as follows: *R*_*x*_ = (50 − 10 × *V*_0_)/*V*_0_. Compared with the traditional wired transmission, the wireless sensation system is compact and portable with the accurate and real-time signal acquisition (Video S1).

With high sensitivity, short response time, and reliable mechanical properties, the poly(ionic liquid) wireless sensor promotes a promising candidate of wearable electronics to identify various body movements and physiological signals. In Figure 6(a), the poly(ionic liquid) hydrogel wireless sensor is fixed on the calf muscle to monitor the leg muscle movement during walking and running. The relative resistance changes vary sharply during walking and running, suggesting repeatable and frequency-dependent muscular activity. Compared to walking, running is accompanied by more vigorous calf muscle activity and a faster speed. The precise monitoring of muscle activity provides reliable data support for sports health monitoring and sports posture correction. The poly(ionic liquid) hydrogel sensor is attached to the knee to monitor different kinds of movement status (Figures 6(c), 6(d), and 6(f)). As shown in Figure 6(c), two consecutive characteristic peaks appear when jumping, corresponding to the first bending up to accumulate energy and a second bending cushioning at landing, relatively. Furthermore, the number of steps, stride frequency, and exercise intensity can also be accurately distinguished. As shown in Figures 6(d) and 6(f), knee movements at running and walking are compared. It can be seen that running shows faster frequency (1.3 s per step) and higher intensity than walking (2 s per step). Meanwhile, the number of steps, distance, and speed of the movement can be obtained under exquisite intensity differentiation and frequency calculation. Therefore, wireless strain sensors show huge market potential in the fields of motion perception, reduction of exercise injuries, and rehabilitation after exercise injuries.

Except for real-time monitoring of leg movements, the poly(ionic liquid) hydrogel wireless strain sensor can also capture wrist movements and finger gestures. In Figure 6(e), the hydrogel wireless strain sensor is attached to the wrist, and the angle of wrist bending is recorded, which provides technical support for remote wrist control and wrist injury rehabilitation. Furthermore, the five-finger knuckles are, respectively, fixed with poly(ionic liquid) hydrogel strain sensors to assemble a data glove (Figure 6(g)). The data glove with five independent wireless strain sensors can separately monitor the activity of each finger and realize precise recognition of gestures. As shown in Figure 6(h), data gloves can recognize different gestures and realize the real-time conversion of sign language letters of “N, J, T, E, C, and H” into digital signals, which play a more decisive role in human-machine interaction and artificial intelligence.

## 3. Conclusion

In summary, lignin-enhanced poly(ionic liquid) double-network hydrogel is proposed to perceive multistimuli of strain, pressure, and temperature. The coordination of “soft” and “hard” segments, dynamic reversible coordination, hydrogen bonding, and electrostatic interaction endow the poly(ionic liquid) hydrogel with impressive stretchability (up to 1425%), toughness (over 132 kPa), and cyclic stability. Moreover, benefitting from the high conductivity thermal-sensitive ionic liquid, the poly(ionic liquid) hydrogel sensor also presents a high gauge factor (GF = 1.37), the short response time (198 ms), and excellent thermal sensitivity (0.217°C^−1^) in the body temperature range. Based on its high-sensitivity performance, the hydrogel sensor can accurately monitor various human movements and be assembled into a flexible touch panel to realize smart input. By integrating the hydrogel sensor into a wireless sensation system, body movement can be tracked and identified in real time and remotely. The hydrogel temperature sensor can also be assembled into a body shell temperature indicator as an auxiliary disease diagnosis and treatment strategy. The poly(ionic liquid) hydrogel, which can identify multistimuli of pressure and temperature simultaneously, presents new application prospects for handwriting encryption, electronic skin, human-machine interface, and remote medical healthcare.

## 4. Material and Methods

### 4.1. Materials

1-Vinylimidazole, acrylic acid (AA, 98%), methacryloylchloride (95%), *N,N*′-methylene bisacrylamide (MBA, 99%), and ammonium persulfate (APS, 99%) were purchased from Adamas-beta. Lignin alkaline (CP) was purchased from Energy Chemistry. 1-Bromobutane (98%) was purchased from Shanghai Lingfeng Chemical Reagent Co., Ltd. Ag nanowire suspension (AgNW, 1.5-2.5 *μ*m in length and 30-40 nm in diameter, 10 mg/mL in ethanol) was purchased from Xfnano. Acrylamide (AM, 98%) was purchased from Tokyo Chemical Industry.

### 4.2. Preparation of 1-Vinyl-3-Butylimidazolium Bromide ([VBIM^+^] Br^−^) Ionic Liquid and Vinyl-Modified Lignin

1-Vinyl imidazole (5 g) and 1-bromobutane (5 g) were added to a centrifuge tube and sealed with N_2_ to prevent oxidation. Subsequently, the mixture was placed in a low-temperature reactor at -8°C and stirred for 24 h. 1-Vinyl-3-butylimidazolium bromide ([VBIM^+^] Br^−^) ionic liquid was obtained. 2 g lignin alkali, 8 g sodium carbonate, and 50 mL toluene were added to a round bottom flask to dissolve and disperse uniformly. Then, 10 mL methacryloyl chloride was added to the solution dropwise in the low-temperature reactor at -8°C. Afterward, the mixed solution was placed in a water bath at 30°C, stirred for 12 h, and desiccated by rotary evaporation. The sample was then washed with 10% sodium carbonate solution three times. Finally, the sample was freeze-dried to remove the remaining solvent, and vinyl-modified lignin powder was obtained.

### 4.3. Preparation of Poly(Ionic Liquid) Hydrogels

0.02 g vinyl-modified lignin and a certain amount of [VBIM^+^] Br^−^ ionic liquid were dispersed in 2.5 ml ultrapure water evenly by ultrasonic. Subsequently, 0.25 g AM, 2 mg MBA, 4 mg borax, and 4 mg APS were added to the solution successively. The mixture was placed in an ultrasonic water bath at 70°C and heated for 10 min, and the poly (ionic liquid) single network hydrogel was obtained. The mass ratio of ionic liquid to ultrapure water was 2.4, 4.8, 9.6, 14.4, 19.2, and 24.0 wt%, respectively. 0.4 ml acrylic acid (AA), 0.75 *μ*l Ag NWs, 2 mg MBA, and 4 mg APS were dissolved in 2.5 mL ultrapure water. Subsequently, the hydrogel was placed in the above solution to swell for 10 min and then taken out. The second radical polymerization reaction was carried out at 60°C for 30 min, and the double-network poly(ionic liquid) hydrogel was obtained.

### 4.4. Mechanical and Electrical Performance Characterization

The operating platform (ESM302, MARK-10) and force gauge (M4-2) were used to operate, record, and analyze the mechanical properties of the hydrogel samples. For the tensile test, the hydrogel sample (10 × 4 × 2 mm) was fixed on the operating platform (ESM302, MARK-10) and performed at a speed of 60 mm/min. For the compression test, a cylindrical hydrogel sample (*ϕ*24 × 14 mm) was prepared and compressed at 10 mm/min. A semiconductive analyzing facility (Keithley 2602) was used to test the electrical properties of the hydrogel samples. The hydrogel samples were inserted into copper wires and fixed on the operating platform (ESM302, MARK-10) as described above. The piezoresistive performance of the hydrogel was acquired on the testing platform, and the analyzing facility recorded the current changes in real time. For body movements and physiological activity detection, the hydrogel sensor was fixed on the specific body position by medical tape, and the electrical signals were tracked. To fabricate a flexible touch panel, the hydrogel sensor was sandwiched between two pieces of flexible film to collect mechanical signals and convert them into an electric signal, a piece of sensitive tape was adhered at the topmost to support and encapsulate the hydrogel, and the VHB tape served as the flexible substrate.

### 4.5. Performance Evaluation of Water Loss

The hydrogel samples were placed in the ambient environment for water loss test at 20°C and RH = 66%. The water loss of the PIL hydrogel was defined as follows:

(1) $$\begin{array}{c} Q=\frac{W_{0}-W_{t}}{W_{0}}\times 100\%, \end{array}$$

where *W*_0_ was the initial weight of the PIL hydrogel and *W*_*t*_ was the weight of the hydrogel after the dehydration experiment.

### 4.6. Thermosensitive Performance Assessment

Cylindrical hydrogel (*ϕ*24 × 6 mm) was connected with copper wires at each end and then encapsulated with PU medical film tape to prevent water loss and external interference. The obtained hydrogel strain sensor was then sealed in a ziplock bag and immersed in a water bath. A series of thermosensitive tests were realized by changing the temperature of the water bath. For simulated body fever and antipyretic process monitoring, infrared cameras (FLIR, ARINGTON, VA) were serviced to record the forehead temperatures.

## Data Availability

All data needed to evaluate the conclusions in the paper are presented in the paper and/or the Supplementary Materials. Additional data related to this paper may be requested from the authors.

## Supplementary Materials

Supplementary Materials Supplementary 1. Figure S1: stretched picture after crossing, knotting, and twisting. Figure S2: 10 consecutive compression-recovery curves of poly(ionic liquid) hydrogel. Figure S3: poly(ionic liquid) hydrogel adhered to PTFE, stone, and metal. Figure S4: pressure-compression curves of hydrogels with different lignin content. Figure S5: scanning electron micrograph of AgNWs. Figure S6: water loss of hydrogel in ambient environment for 24 h (20°C, RH = 66%). Figure S7: circuit diagram of the flexible touch panel. Figure S8: (a) relative current changes when frowning. (b) Relative current changes when fingers were bent at 45° and 90°.
