# Supplementary material for: Thermoresponsive Lignin-Reinforced Poly(Ionic Liquid) Hydrogel Wireless Strain Sensor
Source: Research (Wash D C). 2021 Dec 7;2021:9845482. doi: 10.34133/2021/9845482 (PMC8674648; doi:10.34133/2021/9845482)
Supplement: Supplementary Materials — Supplementary 1. Figure S1: stretched picture after crossing, knotting, and twisting. Figure S2: 10 consecutive compression-recovery curves of poly(ionic liquid) hydrogel. Figure S3: poly(ionic liquid) hydrogel adhered to PTFE, stone, and metal. Figure S4: pressure-compression curves of hydrogels with different lignin content. Figure S5: scanning electron micrograph of AgNWs. Figure S6: water loss of hydrogel in ambient environment for 24 h (20°C, RH = 66%). Figure S7: circuit diagram of the flexible touch panel. Figure S8: (a) relative current changes when frowning. (b) Relative current changes when fingers were bent at 45° and 90°. [file 9845482.f1.zip › Supporting Info.docx]

Supporting Information

**Thermo-responsive Lignin Reinforced Poly(ionic liquid) Hydrogel Wireless Strain Sensor**

**Xinyu Qu,^1^ Ye Zhao,^1^ Zi’ang Chen,^1^ Siying Wang,^1^ Yanfang Ren,^2^ Qian Wang,^1*^ Jinjun Shao, ^1*^ Wenjun Wang,^2^ Xiaochen Dong ^1*^**

*^1^Key Laboratory of Flexible Electronics (KLOFE) and Institute of Advanced Materials (IAM), School of Physical and Mathematical Sciences, Nanjing Tech University (NanjingTech), Nanjing 211816, China.*

*^2^School of Physical Science and Information Technology, Liaocheng University, Liaocheng 252059, China*

Correspondence should be addressed to Xiaochen Dong; iamxcdong@njtech.edu.cn, Qian Wang; chelseawq@njtech.edu.cn, and Jinjun Shao; iamjjshao@njtech.edu.cn.

**
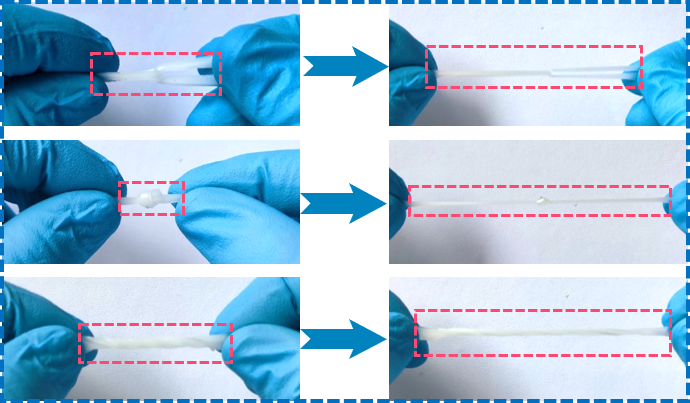
**

Figure S1: Stretched picture after crossing, knotting, and twisting.


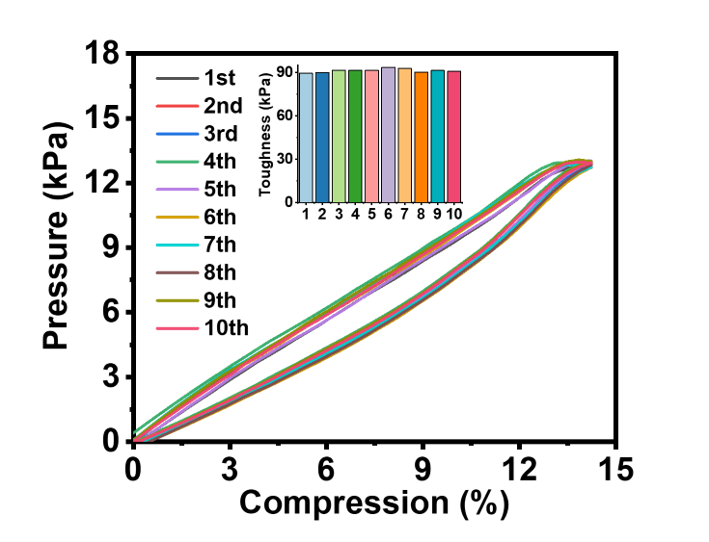


Figure S2: 10 consecutive compression-recovery curves of poly(ionic liquid) hydrogel.


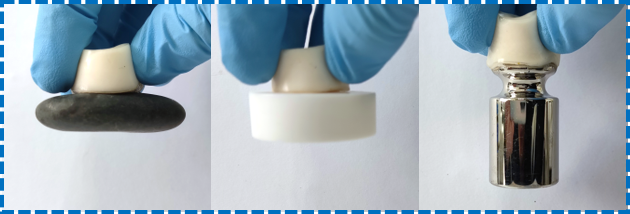


Figure S3: Poly(ionic liquid) hydrogel adhered to PTFE, stone, metal.


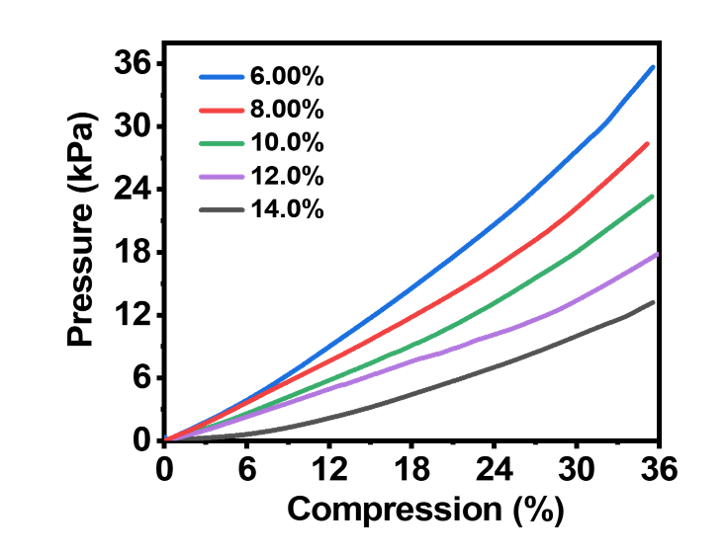


Figure S4: Pressure-compression curves of hydrogels with different lignin content.


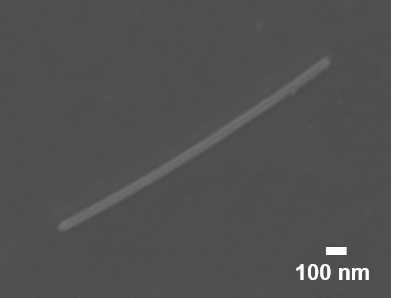

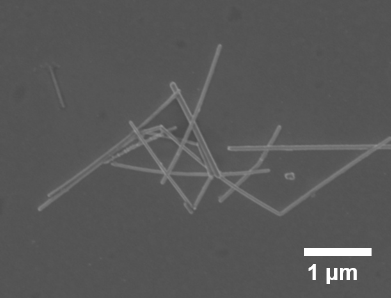


Figure S5: Scanning electron micrograph of AgNWs.


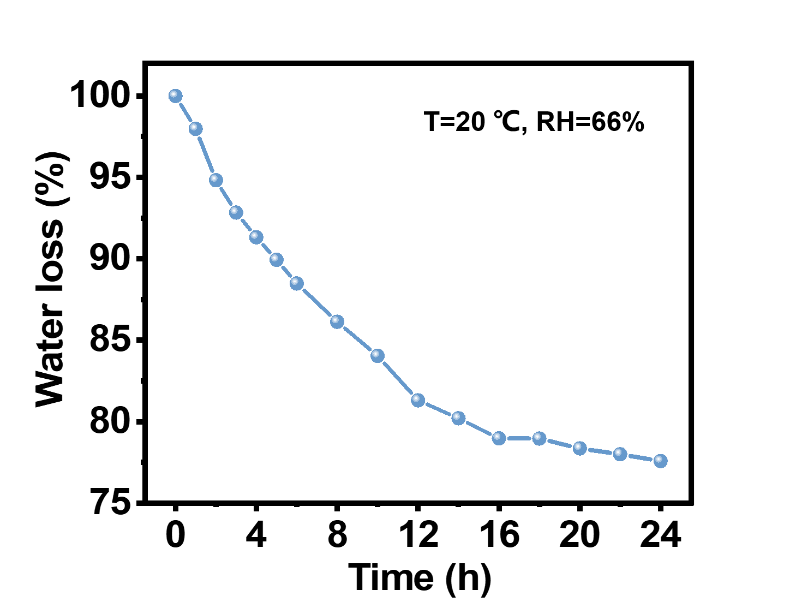


Figure S6: Water loss of hydrogel in the ambient environment for 24 h.


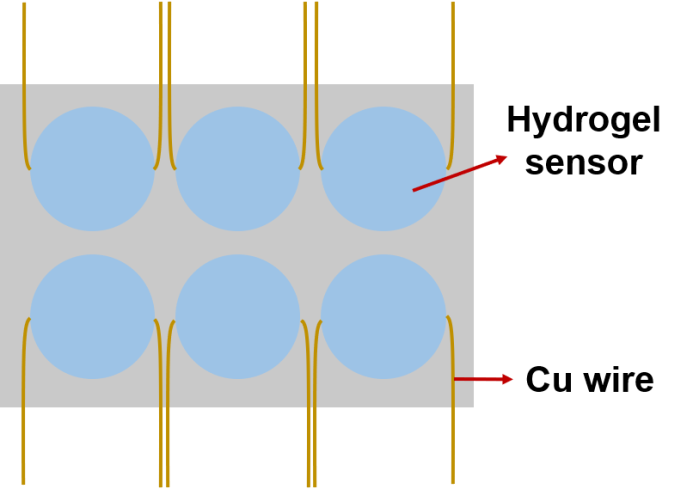


Figure S7: Circuit diagram of the flexible touch panel.


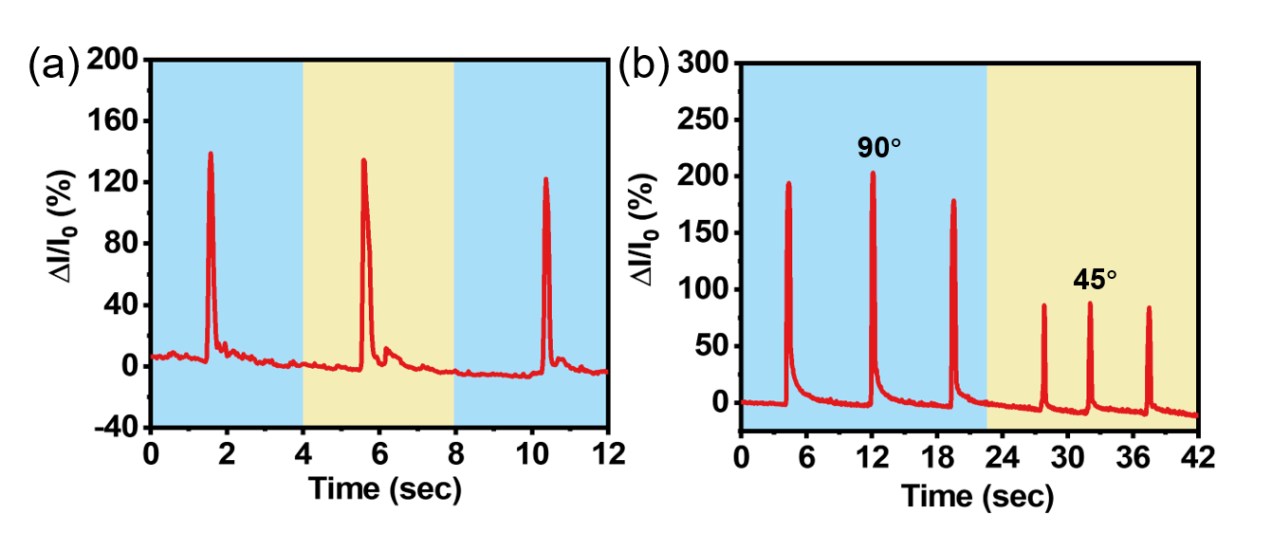


Figure S8: (a) Relative current changes when frowning. (b) Relative current changes when fingers were bent at 45° and 90°.
